# Supplementary material for: Diagnostic accuracy of Panbio rapid antigen tests on oropharyngeal swabs for detection of SARS-CoV-2
Source: PLoS One. 2021 Jun 24;16(6):e0253321. doi: 10.1371/journal.pone.0253321 (PMC8224876; doi:10.1371/journal.pone.0253321)
Supplement: S1 Table — (DOCX) [file pone.0253321.s001.docx]

| S1 Table: Results of the pilot study | | |  |
| --- | --- | --- | --- |
|  | RT-qPCR positive | RT-qPCR negative | Total |
| Panbio^TM^ positive | 11 | 0 | 11 |
| Panbio^TM^ negative | 17 | 28 | 45 |
| Total | 28 | 28 | 56 |
| Sensitivity | 39.28% (95% CI = 21.5 - 59.4%) | |  |
| Specificity | 100% (95% CI = 87.7 - 100%) | |  |
