# Supplementary material for: Diagnostic accuracy of Panbio rapid antigen tests on oropharyngeal swabs for detection of SARS-CoV-2
Source: PLoS One. 2021 Jun 24;16(6):e0253321. doi: 10.1371/journal.pone.0253321 (PMC8224876; doi:10.1371/journal.pone.0253321)
Supplement: S2 Table — (DOCX) [file pone.0253321.s002.docx]

| S2 Table. Patient Characteristics | |
| --- | --- |
| Mean age (±SD, median, range) | 39.9 (±14.5, 38, 16-80) |
| Sex distribution, n (%) |  |
| Women | 224 (55.7) |
| Men | 178 (44.3) |
| Mean days from symptom onset to PCR (SD, Median, range) (n=141) | 4.1 (±3.3, 3, 0-24) |
| Comorbidities among positive PCR, n (%) | 38 (22.6) |
| Number of patients with symptoms, n (%) | 168 (41.8) |
| Symptoms, n (%) (n=168) (descending order in frequencies) |  |
| Asthenia | 101 (60.1) |
| Headaches | 99 (58.9) |
| Myalgia | 81 (48.2) |
| Chills or fever | 80 (47.6) |
| Dry or productive cough | 73 (43.5) |
| Anosmia, agueusia | 71 (42.3) |
| Odynophagia | 68 (40.5) |
| Digestive signs | 38 (22.6) |
| Dyspnea | 7 (4.2) |
| Chest pain | 4 (2.4) |
| Other | 12 (7.1) |
| Had a contact with positive cases within last 14 days, n (%) | 87 (51.8) |
